# Evolution of in-hospital patient characteristics and predictors of death in the COVID-19 pandemic across four waves: are they moving targets with implications for patient care?

**DOI:** 10.3389/fpubh.2023.1280835

**Published:** 2024-01-08

**Authors:** Enrico Maria Trecarichi, Vincenzo Olivadese, Chiara Davoli, Salvatore Rotundo, Francesca Serapide, Rosaria Lionello, Bruno Tassone, Valentina La Gamba, Paolo Fusco, Alessandro Russo, Massimo Borelli, Carlo Torti, the IDTM UMG COVID-19 Group

**Affiliations:** ^1^Department of Medical and Surgical Sciences, “Magna Graecia” University, Catanzaro, Italy; ^2^Infectious and Tropical Disease Unit, “Renato Dulbecco” Teaching Hospital, Catanzaro, Italy; ^3^UMG School of PhD Programmes "Life Sciences and Technologies", “Magna Graecia” University, Catanzaro, Italy; ^4^Dipartimento di Scienze di Laboratorio e Infettivologiche, Fondazione Policlinico Universitario “A. Gemelli” IRCCS, Rome, Italy; ^5^Dipartimento di Sicurezza e Bioetica, Università Cattolica del Sacro Cuore, Rome, Italy

**Keywords:** SARS-CoV-2, COVID-19, pandemic waves, real-world data, public health, kidney disease

## Abstract

**Objectives:**

The aim of this work was to study characteristics, outcomes and predictors of all-cause death in inpatients with SARS-CoV-2 infection across the pandemic waves in one large teaching hospital in Italy to optimize disease management.

**Methods:**

All patients with SARS-CoV-2 infection admitted to our center from March 2020 to June 2022 were included in this retrospective observational cohort study. Both descriptive and regression tree analyses were applied to identify factors influencing all-cause mortality.

**Results:**

527 patients were included in the study (65.3% with moderate and 34.7% with severe COVID-19). Significant evolutions of patient characteristics were found, and mortality increased in the last wave with respect to the third wave notwithstanding vaccination. Regression tree analysis showed that in-patients with severe COVID-19 had the greatest mortality across all waves, especially the older adults, while prognosis depended on the pandemic waves in patients with moderate COVID-19: during the first wave, dyspnea was the main predictor, while chronic kidney disease emerged as determinant factor afterwards.

**Conclusion:**

Patients with severe COVID-19, especially the older adults during all waves, as well as those with moderate COVID-19 and concomitant chronic kidney disease during the most recent waves require more attention for monitoring and care. Therefore, our study drives attention towards the importance of co-morbidities and their clinical impact in patients with COVID-19 admitted to hospital, indicating that the healthcare system should adapt to the evolving features of the epidemic.

## 1 Introduction

As of May 2023, there have been more than 700 million confirmed cases of COVID-19, including almost 7 million deaths reported by the World Health Organization (WHO) globally. According to the Italian National Surveillance System, since the beginning of the COVID-19 pandemic until April 2022, more than 26 million cases of SARS-CoV-2 infection were diagnosed (1). In Italy, the pandemic spread from the northern regions throughout all the Country, but a huge heterogeneity in incidence and fatality rates were reported since the first wave of the pandemic (2–8), evolving towards a lower number of cases and reduced severity of COVID-19. However, since SARS-CoV-2 infections continue to be reported and a recrudescence of the pandemic could occur, it is important to look back to the evolution of the pandemic to get lessons to better manage our patients.

Among the factors which contributed substantially to reduce the incidence and lethality rates, COVID-19 vaccination represented a turning point in the evolution of the pandemic (9). However, outcome of patients varied not only by vaccination status, but also in response to the evolution of SARS-CoV-2 variants and subvariants, adaptation of the health services, new treatments introduced into clinical practice, improved understanding of the disease, skills and expertise of clinicians and patient characteristics in terms of age, sex and comorbidities. Moreover, because all these factors may vary among hospitals and regions, differences in patient outcomes are expected (5).

Several studies analyzed clinical characteristics and outcomes of patients admitted due to COVID-19 across the first three waves in Italy. These studies included mainly unvaccinated individuals showing an improvement in mortality, which paralleled a decrease in the median age (2–4, 6, 8, 10). Similar data were found elsewhere in Europe (11–13). However, to the best of our knowledge, only a retrospective, single center study conducted in Sardinia (Italy) focused on clinical characteristics and outcomes of patients admitted during the fourth wave, observing that patients admitted during this one were older but experienced a milder disease and had a lower risk of admission to the intensive care units (ICU) rather than patients admitted during the previous waves of pandemic, although no data on the vaccination status of these patients were reported (14).

Outcome prediction for a complex disease such as COVID-19 is limited by incomplete knowledge of the relationship among factors that are involved in this disease, therefore leading to possible underestimation of the effect of some factors as mediators [i.e., table 2 fallacy (15)], as well as by lack of consideration of the geographic variability and evolution of the disease overtime, as demonstrated in a recent multicenter Italian study (5). Indeed, survival analysis is usually approached by using Cox models, but this method relies on hazards proportionality assumptions, which for instance are violated when adding an additional mediator (16). In order to overcome the aforementioned difficulty, we tried to focus on a possible different view to establish a hierarchical classification within study covariates, resorting to the conditional tree regression technique (17) as a companion of classical survival analysis. In particular, as it will be discussed thereafter in the introduction, characteristics of the hospitalized patients may evolve and this may have a significant impact on patient outcomes. Unfortunately, the current analyses have studied predictors using a methodology that may incur into possible biases. Therefore, we decided to use the conditional tree regression technique to study predictors of in-hospital deaths in COVID-19 patients along the pandemic waves to try to inform healthcare professionals about the main problems to be taken into account in the more recent stages of the epidemic.

For these reasons, in the present study we aimed at: (*i*) describing clinical characteristics and outcomes of patients admitted to our ward during the four COVID-19 waves; (*ii*) comparing clinical characteristics and outcome of patients across the four pandemic waves; (*iii*) analyzing risk factors, in hierarchical terms, for in-hospital mortality in the entire cohort of our COVID-19 patients taking into account possible differences across the four pandemic waves. In particular, rather than obtaining an explanatory multivariable model or a score to predict the clinical outcome, the goal of this analysis was to explore the most important factors that can increase the risk of in-hospital death across the four pandemic waves.

## 2 Materials and methods

### 2.1 Time frames of the study (waves)

**Figure 1:**
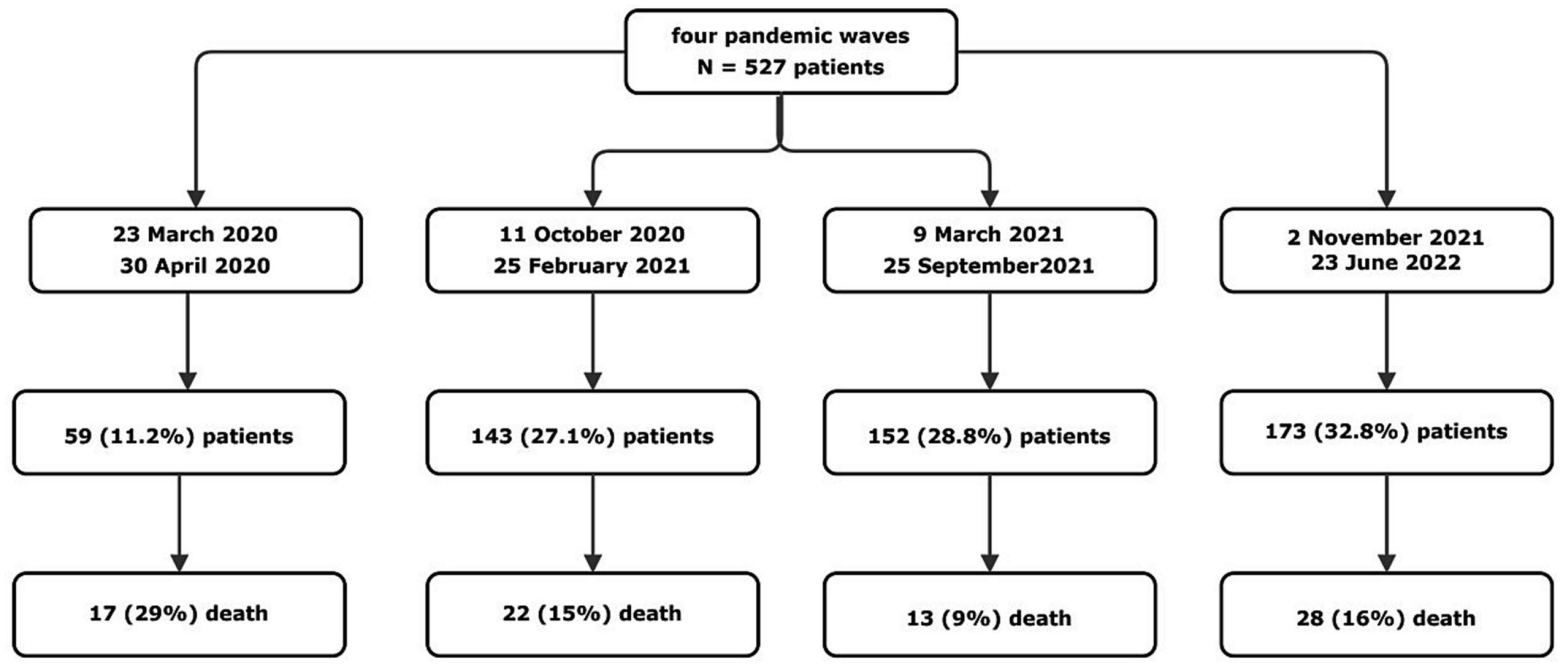
Time frames and study design. A total of 527 patients were included in the study period. The period of time that we considered as pandemic wave are than specified, with the total number of patients admitted and the number of patients died in each wave (Catanzaro, Italy, 2020–2022).

Up until now, there are no universal definitions of “*pandemic wave*.” The WHO only stated that one wave ends when the virus is brought under control and cases drop substantially; for a second wave to start, a sustained rise in infections is needed (18). Based on this definition and consistently with data from Italian Surveillance System (1), we defined four pandemic waves as follows: the first from March 23rd, 2020 to April 30th, 2020; the second one from October 11th, 2020 to February 25th, 2021; the third from March 09th, 2021 to September 25th, 2021; the fourth from November 2nd, 2021 to June 23rd, 2022 (Figure 1).

### 2.2 Setting and data collection

In the present retrospective observational cohort study, all consecutive adult patients infected with SARS-CoV-2 admitted from of March 23rd, 2020 to June 23rd, 2022 to the COVID-19 ward of Infectious and Tropical Diseases Unit of the Teaching Hospital *“Renato Dulbecco”* of Catanzaro in Southern Italy were included as continuation of a previously published study (7). In all these patients SARS-CoV-2 infection was confirmed through molecular testing (i.e., positive SARS-CoV-2 real-time polymerase chain reaction [RT-PCR, GeneFinder COVID-19 Plus RealAmp Kit, Elitech Group] on nasopharyngeal swab), according to WHO recommendations (19).

As established by the hospital directorate of our center, during the first and the second pandemic waves all patients with confirmed SARS-CoV-2 infection were admitted to our ward, including those without significant symptoms attributed to COVID-19, while during the third and the fourth pandemic waves only patients with significant symptoms of COVID-19 or other infectious diseases were admitted to our ward.

For each patient, the following data were collected on the same day of hospital admission and retrospectively retrieved from clinical records for the sake of this study: demographics, clinical and laboratory findings, comorbidities, COVID-19 diagnosis date, home therapies, therapies administered during hospital stay, need for and type of oxygen therapy (i.e., low flow-oxygen, high-flow oxygen and non-invasive and/or invasive ventilation support), length of hospital stay, admission to ICU and in-hospital all-cause death. Within 72 h from admission patients were ranked by WHO clinical scale (19) into two groups: moderate (WHO 4–5) or severe (WHO 6–9) COVID-19. All patients were followed until discharge, end of the study period or death.

The study was approved by the ethical committee of the Calabria Region (protocol reference: FESR/FSE 2014-2020 DDRC n. 4585, Action 10.5.12, noCOVID19@UMG), and it was carried out in accordance with the declaration of Helsinki and the principles of the good clinical practice guidelines (20).

### 2.3 Statistical analysis

We summarized the features and values for continuous and categorical data as number and percentages. Categorical data were compared using chi-square test, while associations in contingency tables have been addressed by the *n*-sample test for equality of proportions, with continuity correction; association within binomial categorical variables has been addressed by Fisher’s exact test for count data. Differences between continuous normally or not-normally distributed covariates have been tested by means of t test, or by Mann–Whitney–Wilcoxon rank sum test with continuity correction, accordingly.

In order to identify a plausible hierarchy within mortality prognostic factors, a multivariable exploratory analysis has been conducted by means of conditional regression trees as implemented in the party package (17). Conditional regression tree is a statistical model that regresses the distribution of a response variable on the status of multiple covariates and offers a more comprehensible representation of the mechanism of the data generating process, especially when heterogeneity of effect measures across covariate levels complicates the separation of direct and indirect effects (15). Evidences obtained by hierarchical analysis were consequently investigated by survival analysis: binary covariates have been addressed by means of the log rank test, as implemented in the survival package (21). To provide confirmation of the significant prediction of the study variables as a possible means to validate our cohort data against the results of the current literature we also performed Cox regression analyses limited to univariate models and adjustment of each variable for age (bivariate models), while multivariable analysis was not conducted to prevent misleading interpretation of secondary risk factors alongside the estimate effect of the primary exposure (15). Age was included in the bivariate models since it consistently emerged as a very important predictor of death (2–4, 6, 7, 14). Associations were expressed using hazard ratios (HRs) with their 95% confidence intervals (CIs). In all inferential instances an alpha = 0.05 significance level has been assumed.

The statistical analysis was performed partly on STATA (a general-purpose Statistical Software Package developed by SataCorp) version 16.1 and partly on the R language (22). The dataset and the R coding are publicly available on GitHub at the repository http://bit.ly/3s9wvm6.

## 3 Results

### 3.1 Patient characteristics and outcomes

A total of 527 patients were included in the study: 59 (11.2%) patients were admitted during the first wave, 143 (27.1%) patients during the second, 152 (28.8%) during the third, and 173 (32.8%) during the fourth. Of these patients 344 (65.3%) had moderate COVID-19 and 183 (34.7%) were affected by severe infection according to the WHO criteria (19).

Demographic, clinical characteristics and outcome (i.e., all cause death) of COVID-19 patients included in the study according to pandemic waves are shown in Table 1. Overall, 299/527 (56%) patients were males, and the mean age (standard deviation, SD) was 67.8 (15.7) years.

**Table 1:** Demographic and clinical characteristics and outcome of COVID-19 patients admitted during the four pandemic waves.

Variables	Total	First wave	Second wave	Third wave	Fourth wave	Overall *p*-values
	(*n* = 527)	(*n* = 59)	(*n* = 143)	(*n* = 152)	(*n* = 173)	
Male sex (*N*, %)	299 (56%)	27 (46%)	83 (52%)	84 (55%)	105 (61%)	0.2404
Age (years, mean ± SD)	67.8 ± 15.7	77 ± 15	66.8 ± 15.4	63.4 ± 14.7	69.7 ± 15.8	<0.001
Length-of-Stay (days, mean ± SD)	15.4 ± 10.8	21.8 ± 12	14.6 ± 10.1	14.5 ± 10.3	14.7 ± 10.9	<0.001
Cancer (*N*, %)	78 (14%)	8 (14%)	14 (10%)	7 (5%)	49 (28%)	<0.001
Hypertension (*N*, %)	334 (63%)	38 (64%)	90 (63%)	88 (58%)	118 (68%)	0.2905
CVD^a^ other than hypertension (*N*, %)	190 (36%)	25 (42%)	39 (27%)	38 (25%)	88 (51%)	<0.001
Psychiatric disorders (*N*, %)	71 (13%)	16 (27%)	14 (10%)	20 (13%)	21 (12%)	0.0732
Neurologic diseases (*N*, %)	116 (22%)	27 (46%)	25 (17%)	18 (12%)	46 (27%)	<0.001
Chronic pulmonary disease (*N*, %)	86 (16%)	12 (20%)	26 (18%)	14 (9%)	34 (20%)	0.0311
Diabetes mellitus (*N*, %)	144 (27%)	14 (24%)	37 (26%)	31 (20%)	62 (36%)	0.0162
Chronic kidney disease (*N*, %)	105 (19%)	21 (36%)	16 (11%)	10 (7%)	58 (34%)	<0.001
Obesity (*N*, %)	172 (32%)	8 (14%)	50 (35%)	50 (33%)	64 (37%)	0.0052
COVID-19 symptoms (*N*, %)	445 (84%)	51 (86%)	122 (85%)	140 (92%)	132 (72%)	0.0847
Fever (*N*, %)	287 (54%)	41 (69%)	96 (67%)	91 (60%)	59 (34%)	0.0326
Cough (*N*, %)	234 (44%)	21 (36%)	66 (46%)	67 (44%)	80 (46%)	0.0426
Dyspnoea (*N*, %)	231 (43%)	12 (20%)	66 (46%)	79 (52%)	74 (43%)	0.0003
Diarrhoea (*N*, %)	20 (3%)	0 (0%)	4 (3%)	11 (7%)	5 (3%)	NA
Asthenia (*N*, %)	159 (30%)	3 (1%)	56 (39%)	52 (34%)	48 (28%)	<0.001
Number of medications before admission (mean ± SD)	4.5 ± 3.8	5.4 ± 3.5	3.9 ± 3.4	3.4 ± 3.5	5.8 ± 4.2	<0.001
Oxygen therapy (*N*, %)	378 (71%)	21 (36%)	116 (81%)	137 (90%)	104 (60%)	<0.001
Low-flow oxygen (*N*, %)	195 (37%)	16 (27%)	60 (42%)	77 (51%)	42 (35%)	<0.001
High-flow oxygen (*N*, %)	65 (12%)	3 (5%)	17 (12%)	23 (15%)	22 (13%)	<0.001
Non-invasive ventilation (*N*, %)	71 (13%)	0 (0%)	21 (15%)	21 (14%)	29 (17%)	NA
Oro-tracheal intubation (*N*, %)	47 (9%)	2 (3%)	18 (13%)	16 (11%)	11 (6%)	<0.001
Anticoagulant drugs (*N*, %)	487 (92%)	58 (98%)	141 (99%)	146 (96%)	142 (82%)	0.0023
Enoxaparin (*N*, %)	446 (84%)	54 (92%)	139 (97%)	141 (93%)	112 (65%)	<0.001
Fondaparinux (*N*, %)	45 (8%)	7 (12%)	13 (9%)	9 (6%)	16 (9%)	0.0023
Antibiotics (*N*, %)	379 (71%)	55 (93%)	115 (80%)	100 (66%)	109 (63%)	<0.001
Azithromycin (*N*, %)	102 (19%)	49 (83%)	44 (31%)	3 (2%)	6 (3%)	<0.001
Ceftriaxone (*N*, %)	210 (39%)	5 (8%)	95 (66%)	79 (52%)	31 (18%)	<0.001
Corticosteroid therapy (*N*, %)	384 (72%)	36 (62%)	121 (85%)	131 (86%)	96 (55%)	<0.001
Remdesivir therapy (*N*, %)	166 (31%)	0 (0%)	65 (45%)	68 (45%)	33 (19%)	<0.001
Tocilizumab (*N*, %)	30 (5%)	5 (8%)	8 (6%)	7 (5%)	10 (6%)	0.1912
Remdesivir short course^b^ (*N*, %)	39 (7%)	0 (0%)	0 (0%)	0 (0%)	39 (23%)	NA^e^
Monoclonal antibodies (*N*, %)	85 (16%)	0 (0%)	0 (0%)	15 (10%)	70 (40%)	NA^e^
Vaccination (*N*, %)	133 (25%)	0 (0%)	0 (0%)	9 (6%)	124 (72%)	NA^e^
WHO^c^ scale 4–5 (*N*, %)	344 (65%)	54 (92%)	87 (61%)	92 (61%)	111 (64%)	<0.001
WHO^c^ scale 6–9 (*N*, %)	183 (34%)	5 (8%)	56 (39%)	60 (39%)	62 (36%)	<0.001
ICU^d^ stay (*N*, %)	67 (12%)	2 (3%)	23 (16%)	20 (13%)	22 (13%)	<0.001
Death (*N*, %)	80 (15%)	17 (29%)	22 (15%)	13 (9%)	28 (16%)	0.0038

Overall *p*-values reported, respectively, refers to Fisher exact test for contingency table or to Kruskal–Wallis rank sum test, according to counts or continuous data for all the four waves (i.e., Age and Length-of-Stay) (Catanzaro, Italy, 2020–2022). SD, standard deviation; ^a^CVD, cardiovascular disease; ^b^Remdesivir short course, early treatment with remdesivir; ^c^WHO, world health organization scale of COVID-19 severity; ^d^ICU, intensive care unit; ^e^NA, Not Applicable.

The overall in-hospital mortality rate in our cohort was 15% (80/527 patients) and it significantly differed along the four COVID-19 waves, ranging from 8.6% during the third wave to 15.4% during the second one, 16.2% during the fourth one, and 28.8% during the first one (*n*-sample test for equality of proportions with continuity correction, *p* = 0.003). Since increasing in-hospital mortality during the fourth wave was noticed in contrast with its progressive decrease during the first three waves, we focused on differences in clinical characteristics of patients admitted during this last wave compared to the previous ones.

Compared to those belonging to the first three waves (Table 2), patients admitted during the fourth wave were affected more frequently by cancer (*p <* 0.001), cardiovascular diseases other than hypertension (*p* < 0.001), diabetes mellitus (*p* = 0.002) and/or chronic kidney disease [CDK, defined as estimated glomerular filtration rate below 60 mL/min (23), *p* < 0.001], received hospital therapy with factor Xa/direct thrombin inhibitors or underwent early COVID-19 treatment with short course remdesivir (i.e., for three days) and/or monoclonal antibodies against SARS-CoV-2 (*p* < 0.001). Furthermore, patients admitted during the fourth wave were taking a greater number of medications due to comorbidities compared to those in the previous waves (*p* < 0.001).

**Table 2:** Comparison of demographic and clinical characteristics and outcome of COVID-19 patients admitted in the fourth pandemic wave vs. those admitted during the first three waves.

Variables	First-third waves	Fourth wave	OR (wave 4 *vs* waves 123)	95% IC	*p-*values
	(*n* = 354)	(*n* = 173)			
Male sex (*N*, %)	194 (55%)	105 (61%)	0.79	0.53–1.16	0.19
Age (years, mean ± SD)	67.0 ± 15.7	69.7 ± 15.8	–	–	0.07
Cancer (*N*, %)	29 (8%)	49 (28%)	4.43	2.6–7.6	<0.001
Hypertension (*N*, %)	216 (61%)	118 (68%)	1.37	0.92–2.06	0.11
CVD^a^ other than hypertension (*N*, %)	102 (29%)	88 (51%)	2.56	1.72–3.79	<0.001
Chronic pulmonary disease (*N*, %)	52 (15%)	34 (20%)	1.42	0.85–2.34	0.15
Diabetes mellitus (*N*, %)	82 (23%)	62 (36%)	1.85	1.22–2.81	0.002
Chronic kidney disease (*N*, %)	47 (13%)	58 (34%)	3.29	2.07–5.24	<0.001
Obesity (*N*, %)	108 (31%)	64 (37%)	1.34	0.89–1.99	0.14
COVID-19 symptoms (*N*, %)	313 (88%)	132 (76%)	0.42	0.25–0.70	0.0003
Fever (*N*, %)	228 (64%)	59 (34%)	0.29	0.19–0.43	<0.001
Number of medications before admission (mean ± SD)	3.9 ± 3.5	5.8 ± 4.2	–	–	<0.001
Oxygen therapy (*N*, %)	274 (77%)	104 (60%)	0.44	0.29–0.66	<0.001
Low-flow oxygen (*N*, %)	153 (43%)	42 (35%)	0.42	0.27–0.64	<0.001
High-flow oxygen (*N*, %)	43 (12%)	22 (13%)	1.05	0.57–1.87	0.85
Non-invasive ventilation (*N*, %)	42 (12%)	29 (17%)	1.49	0.86–2.56	0.12
Oro-tracheal intubation (*N*, %)	36 (10%)	11 (6%)	0.59	0.26–1.24	0.14
Anticoagulant drugs (*N*, %)	345 (97%)	142 (82%)	0.12	0.05–0.27	<0.001
Enoxaparin (*N*, %)	334 (94%)	112 (65%)	0.11	0.06–0.2	<0.001
Fondaparinux (*N*, %)	29 (8%)	16 (9%)	1.14	0.56–2.25	0.68
Antibiotics (*N*, %)	270 (76%)	109 (63%)	0.53	0.35–0.8	0.0015
Azithromycin (*N*, %)	96 (27%)	6 (3%)	0.1	0.03–0.23	<0.001
Ceftriaxone (*N*, %)	179 (50%)	31 (18%)	0.21	0.13–0.34	<0.001
Corticosteroid therapy (*N*, %)	288 (81%)	96 (55%)	0.29	0.19–0.44	<0.001
Remdesivir therapy (*N*, %)	133 (38%)	33 (19.1%)	0.39	0.25–0.62	<0.001
Tocilizumab (*N*, %)	20 (6%)	10 (6%)	1.02	0.42–2.36	0.95
Remdesivir short course^b^ (*N*, %)	1 (0,3%)	39 (23%)	102.74	16.9–4171.25	<0.001
Monoclonal antibodies (*N*, %)	15 (4%)	70 (40%)	15.36	8.23–29.98	<0.001
Vaccination (*N*, %)	9 (3%)	124 (72%)	97.01	45.01–228.01	<0.001
WHO^c^ scale 4–5 (*N*, %)	233 (66%)	111 (64%)	0.93	0.62–1.39	0.71
WHO^c^ scale 6–9 (*N*, %)	121 (34%)	62 (36%)	1.08	0.72–1.6	0.71
ICU^d^ stay (*N*, %)	45 (13%)	22 (13%)	1	0.55–1.77	0.1
Death (*N*, %)	52 (15%)	28 (16%)	1.12	0.65–1.89	0.65

*p*-values reported, respectively, refers to Fisher exact test for contingency table or to Kruskal–Wallis rank sum test, according to counts or continuous data. SD, standard deviation; ^a^CVD, cardiovascular disease; ^b^Remdesivir short course, early treatment with remdesivir; ^c^WHO, world health organization scale of COVID-19 severity; ^d^ICU, intensive care unit.

Most patients admitted during the fourth wave received SARS-CoV-2 vaccination (124/173, 71.7%). By contrast, among 354 patients admitted during the first three waves, only nine were vaccinated against SARS-CoV-2 (2.5%). Patients admitted during the first three waves more frequently reported at admission COVID-19-related symptoms (*p* < 0.001), especially fever (*p* < 0.001). Although no statistically significant differences were observed for WHO clinical progression scale scores, patients admitted during the fourth wave were less likely to receive oxygen therapy (*p* < 0.001) in particular as far as low-flow oxygen therapy concerned (*p* < 0.001) and less frequently received remdesivir (*p* < 0.001), corticosteroids (*p* < 0.001), enoxaparin (*p* < 0.001) and/or antibiotics (*p* < 0.001) during the hospital stay.

### 3.2 Conditional regression tree analysis

**Figure 2:**
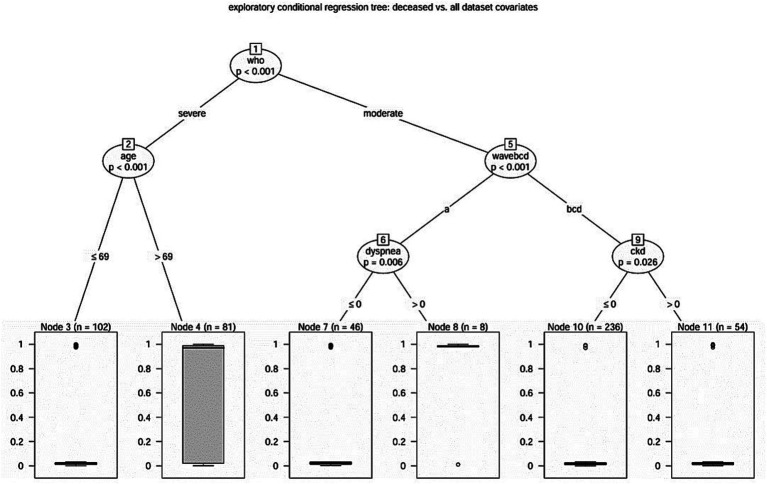
Hierarchical scheme for conditional regression tree analysis between mortality and the overall study covariates (Catanzaro, Italy, 2020–2022).

In order to establish a hierarchy between mortality and the overall study covariates, we resorted to the technique of conditional regression tree (17) (Figure 2). Five covariates had been identified according to the hierarchical scheme exposed in Figure 2, enhancing the main hierarchical role of the WHO classification (moderate versus severe cases), and identifying the proper role of patients age, the different COVID-19 wave and, respectively, the role of dyspnea and of CKD.

A decreased proportion of 7.3% deaths (*n* = 25) in the moderate case group versus 30.1% deaths (*n* = 55) in the severe one (Fisher’s exact test, *p* < 0.001) was found, accounting for an odds ratio of 5.46 (95%CI 3.19–9.57).

Concerning the group of severe COVID-19 patients, according to conditional tree regression analysis, older age revealed to be a significant predictor for mortality. Indeed, patients older than 69 years had a significantly higher proportion of deaths with 55.6% (*n* = 45) in the older patient group versus 9.8% deaths (*n* = 10) in the younger one (Fisher’s exact test, *p* < 0.001), accounting for an odds ratio of 11.32 (95% CI 4.98–27.98).

The regression tree disclosed the different (*p* < 0.001) behavior during the first pandemic wave: with an odds ratio of 0.06 (95% CI 0.02–0.17) we observed a significantly (Fisher’s exact test, *p* < 0.001) different deceased proportion of 17 deaths (31.5%) during the first pandemic wave versus 8 deaths (2.7%) in the three ones. In particular, during the first pandemic wave, 7 over 8 patients (87.5%) who reported dyspnea at admission died (Fisher’s exact test, *p* < 0.001), versus 10 over 46 eupneic patients (odds ratio 23.4, 95% CI 2.56–1157.44). Moreover, patients who reported dyspnea had a median length of stay of 5.5 days, which was significantly shorter from that of those who not reported that symptom who had a median length of stay of 26 days (Log Rank test, *p* < 0.001). Conversely, during the other pandemic waves, CKD appears to play a significant (Fisher’s exact test, *p* = 0.007) role in predicting mortality: with an odds ratio of 7.84 (95% CI 1.47–52.17) 5 casualties in the CKD group (9.3%) were recorded versus the 3 casualties (1.3%) of the not CKD group. Also, in this case the median length of stay was different (Log Rank test, *p* = 0.02) in the two groups: 13 days in the CKD group, 9 days in the not-CKD group.

**Figure 3:**
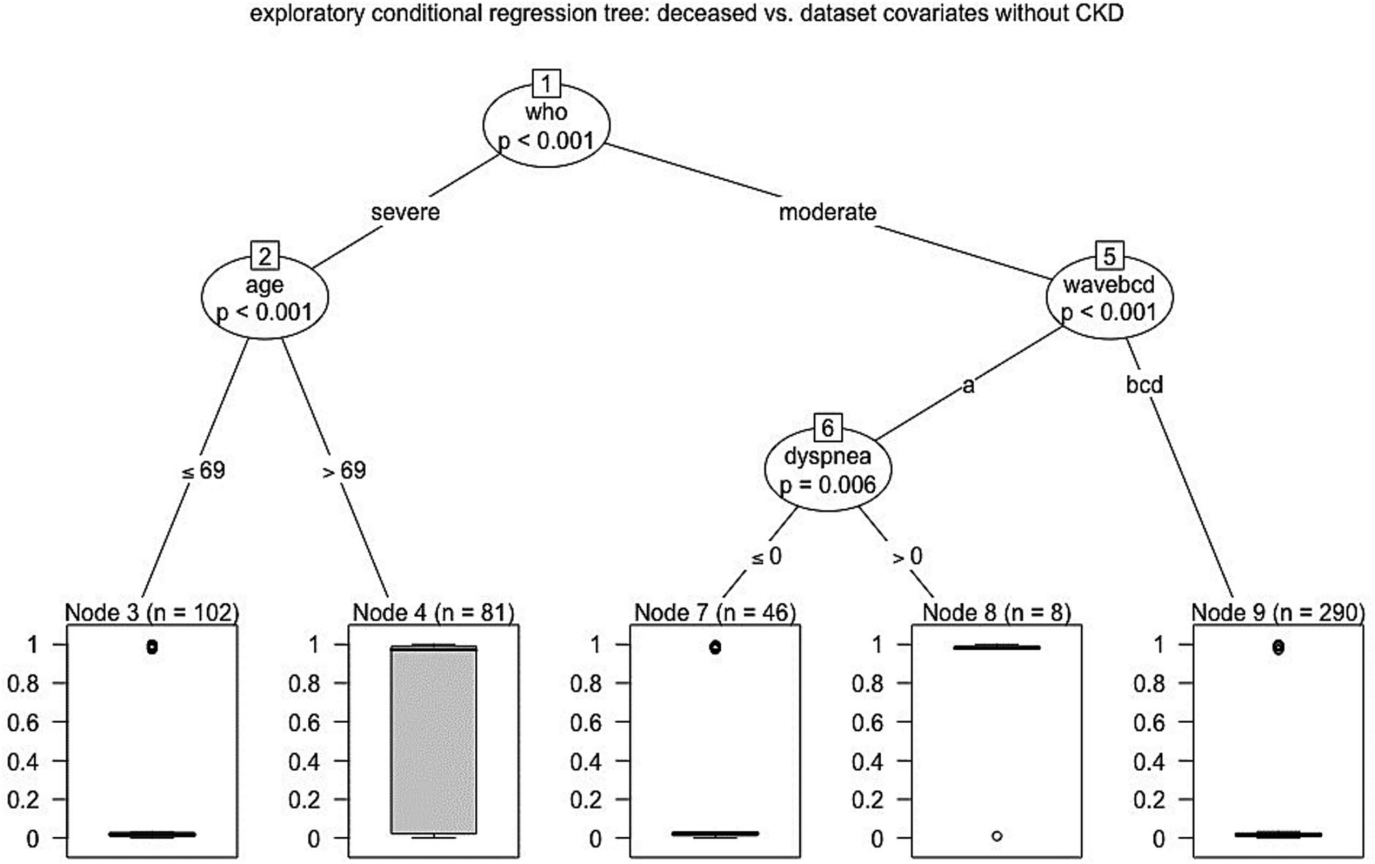
Hierarchical scheme for conditional regression tree analysis between mortality and dataset covariates without CKD (Catanzaro, Italy, 2020–2022).

For completeness, we performed further regression tree analyses. We started investigating what occurred when removing the CKD covariate from the analysis, disclosing that no further predictor appeared in the ninth node. Moreover, when the analysis was repeated in a data subset obtained dropping out the first wave patients, the tree appeared to be simpler, but it again enhanced the significant (*p* < 0.001) role of the WHO classification; in fact, among the severe cases, the 69 years old age or more discriminated poor outcome (*p* < 0.001) as well as it was for CKD (*p* = 0.028) among inpatients with moderate COVID-19 (Figure 3).

### 3.3 Univariate and age-adjusted bivariate Cox regression analyses of risk factors for in-hospital mortality

After exclusion of time-dependent variables, the univariate Cox analysis showed that age (*p* < 0.001), cancer (*p* = 0.02), CVD other than hypertension (*p* < 0.001), chronic pulmonary disease (*p* < 0.001), chronic kidney disease (*p* < 0.001), neurological disease (*p* = 0.006), presence of dyspnea (*p* = 0.001), and a lower level of arterial oxygen saturation at hospital admission (*p* < 0.001) were associated with a higher risk of in-hospital mortality (Table 3). After adjusting for age, only chronic pulmonary disease (*p* = 0.001), chronic kidney disease (*p* = 0.03), presence of dyspnea (*p* < 0.001), and a lower level of arterial oxygen saturation at hospital admission (*p* < 0.001) remained significantly associated with in-hospital mortality; in addition, also hypertension was significantly associated with a higher risk of in-hospital mortality.

**Table 3:** Unadjusted univariate and age-adjusted bivariate Cox analyses for in-hospital mortality in patients admitted during the four pandemic waves (Catanzaro, Italy, 2020–2022).

Variables	Non-survivors	Survivors	Unadjusted HR (95% IC)	*p*-values	Age-adjusted HR (95% IC)	*p*-values
	(*n* = 80)	(*n* = 447)				
Male sex (*N*, %)	45 (56%)	254 (57%)	1.08 (0.69–1.68)	0.74	0.79 (0.50–1.29)	0.32
Age (years, mean ± SD)	78.9 ± 11.9	65.9 ± 15.6	1.06 (1.04–1.08)	<0.001	–	–
Cancer (*N*, %)	18 (23%)	60 (13%)	1.82 (1.08–3.08)	0.02	1.41 (0.82–2.40)	0.20
Hypertension (*N*, %)	53 (66%)	281 (63%)	0.93 (0.59–1.49)	0.77	0.53 (0.33–0.087)	0.01
CVD^a^ other than hypertension (*N*, %)	45 (56%)	145 (32%)	2.35 (1.51–3.66)	<0.001	1.51 (0.95–2.40)	0.07
Psychiatric disorders (*N*, %)	8 (10%)	63 (14%)	0.52 (0.25–1.08)	0.08	0.50 (0.24–1.05)	0.07
Neurologic diseases (*N*, %)	29 (36%)	87 (19%)	1.89 (1.19–2.98)	0.006	1.13 (0.70–1.84)	0.59
Chronic pulmonary disease (*N*, %)	28 (35%)	58 (13%)	2.69 (1.70–4.28)	<0.001	2.26 (1.42–3.59)	0.001
Diabetes mellitus (*N*, %)	28 (35%)	116 (26%)	1.46 (0.92–2.31)	0.11	1.32 (0.83–2.09)	0.23
Chronic kidney disease (*N*, %)	33 (41%)	72 (16%)	2.95 (1.89–4.61)	<0.001	1.70 (1.05–2.78)	0.03
Obesity (*N*, %)	27 (34%)	145 (32%)	0.97 (0.61–1.55)	0.89	1.25 (0.78–2.01)	0.34
Symptoms (*N*, %)	72 (90%)	373 (83%)	1.46 (0.70–3.02)	0.31	1.90 (0.91–3.96)	0.08
Fever (*N*, %)	42 (53%)	245 (55%)	0.85 (0.55–1.32)	0.47	1.08 (0.69–1.69)	0.71
Cough (*N*, %)	33 (41%)	201 (45%)	0.88 (0.56–1.37)	0.57	1.07 (0.68–1.68)	0.32
Dyspnea (*N*, %)	54 (68%)	177 (40%)	2.27 (1.42–3.62)	0.001	2.84 (1.77–4.56)	<0.001
Diarrhea (*N*, %)	4 (5%)	16 (4%)	1.32 (0.48–3.63)	0.58	1.76 (0.63–4.85)	0.27
Asthenia (*N*, %)	19 (24%)	140 (31%)	0.77 (0.46–1.29)	0.31	1.09 (0.64–1.87)	0.72
Oxygen saturation (%, mean ± SD)	89.7 ± 9.4	94.6 ± 5.1	0.95 (0.93–0.97)	<0.001	0.93 (0.91–0.95)	<0.001
Vaccination (*N*, %)	16 (20%)	117 (26%)	0.80 (0.46–1.38)	0.78	0.72 (0.42–1.26)	0.25

SD, standard deviation; ^a^CVD, cardiovascular disease.

## 4 Discussion

Despite WHO established that SARS-CoV-2 infection is no longer a public health emergency of international concern (24), it is still an outstanding problem, therefore clinical management (e.g., home isolation, outpatient therapies, hospitalization) should be tailored by considering predictors of outcome that could change over time for several reasons (4). Many studies investigated predictors of the clinical outcomes, however classical survival analysis approach does not properly fulfill the need to recognize primary from secondary risk factors for the outcome (15), even for a possible presence of a framework of competitive death risks (25). In this study, we were interested to study possible predictors or factors associated with the primary outcome of death across the pandemic waves using a more appropriate method, both to define priorities based on patient characteristics and to suggest more targeted care and use of resources. To the best of our knowledge, the present analysis is the first conducted in a relatively large cohort of real-life patients taking into consideration possible differences among the four waves avoiding possible biases related to the table-2 fallacy and competitive risks. Indeed, we resorted the regression tree analysis technique in order to highlight risk factors associated with survival. It is known that conditional regression tree analysis offers major advantages over other regression models (26), i.e., to handle multicollinearity (which is typical in larger screening “pilot” dataset) in explanatory variables by selecting the best splitter at each node, and its ability to identify “outlier patients” (in our case, the patients cohort during the first pandemic wave).

Results of the present study confirm that patients admitted during the first wave had a very poor prognosis. Indeed, the lack of knowledge on management of COVID-19 and the unpreparedness of health system could have played a pivotal negative role in that period, especially due to the unavailability of both vaccines and effective therapies against SARS-CoV-2 (7). Mortality rate in each of the four waves was relatively high; however, it decreased considerably from the first (29%) to the third wave (9%), in line with previous studies (2, 4, 10–13, 27), but an increase in-hospital mortality rate was found in the fourth wave (16%). To the best of our knowledge this finding was not previously reported in other studies, and it could be explained by the presence of more severe underlying comorbidities in such patients (28, 29), suggesting that these patients could benefit from a multidisciplinary approach that involves several specialists.

Furthermore, we found that patients admitted during the first three waves were mainly treated for COVID-19 pneumonia, in particular with remdesivir (five days course), corticosteroids and enoxaparin, compared to patients admitted during the fourth wave. By contrast, a significantly higher proportion of these latter patients were treated with early COVID-19 therapies (e.g., short course of remdesivir and/or monoclonal antibodies against SARS-CoV-2) compared to patients admitted during the first three waves, thus confirming that, in our experience, during the fourth wave COVID-19 complications (e.g., acute respiratory failure and/or pneumoniae) were not the main reason for patient admission. These findings suggest that the effectiveness of vaccination (30), early antiviral therapies against SARS-CoV-2 (31, 32) and probably the lower pathogenicity of the viral variants (33, 34) changed the clinical needs of patients admitted due to COVID-19 over time and this should be taken into account in relation to the commitment and management of resources.

To avoid misleading interpretation of secondary risk factors, we decided to use regression tree analysis to explore the most important predictors or factors associated with all-cause death. Not unexpectedly, severity of the diseases appeared to be a key factor. In fact, while inpatients affected by severe COVID-19 had a significantly greater risk of death regardless waves of the pandemic, with increasing risk of death depending only on age, those with moderate COVID-19 according to WHO clinical progression scale, showed a different behavior as far as the risk of death was concerned depending on the pandemic waves. Therefore, not only geographical differences reflecting heterogenicity in demographic and clinical characteristics of patients (5), but also the pandemic waves are an important factor which may have an impact on patient prognosis. For these reasons, prognostic scores (including clinical variables, radiological features and biomarkers) that were elaborated in the previous waves (35–40) may change their accuracy across different pandemic waves and should be re-evaluated in the current epidemiological scenario.

Indeed, among patients with moderate COVID-19 admitted during the first wave, dyspnea was the main variable associated with in-hospital mortality. In such cases dyspnea could be considered as a prodromic condition for clinical progression or a proxy of severe COVID-19 that was misclassified as moderate since more intensive ventilatory support (e.g., high-flow oxygen or non-invasive ventilation)—which defines the severity of the disease according to WHO classification (19)—was avoided, not because it was unnecessary, but due to its unavailability in our hospital during the first wave of the pandemic.

Regarding patients with moderate COVID-19 admitted during the subsequent three waves, CKD appeared to be the discriminant factor. In fact, during the last three waves, most inpatients with moderate COVID-19 who died were also affected by CKD. It has been already highlighted that CKD can increase COVID-19 related mortality when compared to non-CKD patients (41), especially before vaccination (42). Besides the fact that patients with CKD may be older, more frequently affected by other comorbidities leading to this complication, and more subjected to drug adverse events, one of the reasons why CKD may be associated with COVID-19 mortality is that SARS-CoV-2 antivirals [except molnupiravir whose marketing authorization was recently refused by European Medical Agency (EMA) (43)] were contra-indicated for patients with an eGFR below 30 mL/min, while they could have improved patient prognosis for a positive effect on COVID-19. Only in July 2023, Food and Drug Administration and EMA approved the use of remdesivir in patients with renal impairment, including patients on dialysis, relying on the REDPINE trial (44), even though there is still a lack of data on safety in patients with severe CKD or end-stage renal disease. We also found that patients who experienced dyspnea had a significantly shorter median length of stay compared to those who did not report that symptom; conversely, median length of stay was significantly longer in the CKD group compared to the not-CKD group. However, these differences in length of stay could be misleading, because the analyses included also patients who died; therefore, patients suffering from dyspnea (which was a main risk factor for mortality during the first wave), could have had a significantly shorter length of stay because they died earlier. Nonetheless, in terms of burden on the healthcare system, the impact of COVID-19 could still be significant.

Lastly, although we are aware of the limitations affecting Cox regression—such that only univariate and bivariate models were performed—the results of these analyses, after adjusting for age, showed that several comorbidities, such as hypertension (*p* = 0.01), chronic pulmonary disease (*p* = 0.001), CKD (*p* = 0.03), and presence of dyspnea (*p* < 0.001), and lower peripheral oxygen saturation (*p* < 0.001) were correlated with an increased risk of death, confirming results of previous works (26, 27, 30, 31) and providing further support to the validity of our cohort.

In summary, the study included 527 patients with COVID-19, of whom 80 died in the hospital. The mortality rate was significantly higher in the first wave of the pandemic compared to the other three waves. Patients admitted during the fourth wave were more likely to have underlying comorbidities, such as cancer, cardiovascular diseases, diabetes, and chronic kidney disease. Univariate and age-adjusted bivariate Cox regression analyses confirmed that chronic pulmonary disease, chronic kidney disease, dyspnea at admission, and lower arterial oxygen saturation at hospital admission were independently associated with an increased risk of in-hospital mortality. Importantly, conditional regression tree analysis brought to light that the following risk factors emerged as the possible main determinants for in-hospital mortality: (i) WHO classification of COVID-19 severity (moderate vs. severe); (ii) age (older than 69 years); (iii) first pandemic wave; (iv) dyspnea at admission (during the first wave); (v) chronic kidney disease (during the waves after the first one). It has to be recognized, however, that the conditional regression tree analysis method used in the study, besides the advantage of having a tree structure easy to visualize, suffers from some potential disadvantages: first, the nested risk factors for mortality could be challenging to interpret; second, the method is sensitive to the clinicians choice of covariates, and may be affected by instability because small changes in the data can lead to possibly large changes in the tree structure and the results; lastly, the study was conducted in a single center, which limits the generalizability of the results.

This study has some limitations that should be discussed. First, this is the first real-life analysis conducted in a relatively large cohort of patients comparing the first four waves of COVID-19 pandemic in Southern Italy and only few data about clinical differences and outcomes among these waves in the Italian population are available. However, the cohort analyzed may lack of representativeness for important factors, such as socio-economic status, education in prevention, time of access to therapy and hospital care or types of clinical management applied. Therefore, the generalizability of our results throughout Italy is limited because there was heterogenicity among the italian regions (1, 5) in terms of demographic characteristics, population density, infection rate, organization of the health-care system and availability of assistance, which in turn may have a significant impact of the study outcomes. Multicentric studies are needed to understand if our results are confirmed in different settings across Italy. Second, despite the fact that there was a lack of data about SARS-CoV-2 variants of concern (VoCs) and subvariants in individual patients, the general picture and evolution are well known in our region since an epidemiological survey coordinated by our center demonstrated that in August 2021, Delta was the only circulating variant, while as of January 2022, Omicron subvariants emerged and took over Delta (45, 46). Therefore, SARS-CoV-2 variants and subvariants do not appear to be a reasonable explanation for the increase of the inpatients mortality during the fourth wave, because Omicron variants/subvariants prevailed in this period, being already associated with reduced virulence, particularly in patients who received full vaccination course (47). Third, following the instructions of our Hospital Directorate, during the first and the second pandemic waves all patients with confirmed SARS-CoV-2 infection were admitted to our ward, while during the third and the fourth pandemic waves only patients with COVID-19 as main problem or patients with SARS-CoV-2 infection suffering from other infectious diseases were admitted to our ward. Therefore, this could influence the interpretation of the comparison between patients admitted during the different waves. Indeed, the net effect on inpatient mortality could be a result of the severity of COVID-19 combined with patients age, number and severity of comorbidities, and the protective immunity against SARS-CoV-2 which is correlated to the number of doses of vaccine received, waning over time since the last dose. It is impossible to weight the effect of each single component for this network of factors, however our objective was rather descriptive than explanatory since we were interested in getting insights on the main problems faced by both patients and clinicians throughout the different COVID-19 pandemic waves, posing a clear rationale to use a descriptive analysis and regression tree methodology. Given the complexity of epidemiological and clinical pictures of COVID-19 pandemic, which also has implied an important impact on economic and social growth (48), knowledge deriving from such an approach could lead to optimization of patient management and organization of the health system.

## 5 Conclusion

This study highlights significant differences in patient characteristics and outcomes across the four COVID-19 waves in our teaching hospital. Using the regression tree analysis, we found that COVID-19 severity (defined by WHO severity scale) was the main predictor of outcome, regardless of the pandemic wave: older patients with severe COVID-19 were always at high risk of death. In patients with moderate COVID-19, our analysis showed that those admitted during the last three pandemic waves and suffering from CKD had the worst outcome. On the other hand, dyspnoea was shown to be the main predictor of mortality in moderate COVID-19 patients admitted during the first wave. Overall, our results suggest that knowledge of the changing characteristics of COVID-19 patients might be helpful to better understand how the provision of the disease will be in the future and what clinical/health needs should be approached for the management of this disease. In particular, despite the fact that patients admitted in the fourth wave were mostly vaccinated and more often suffered from a moderate/mild COVID-19 diseases, they had severe underlying comorbidities and displayed a rate of in-hospital mortality which was significantly higher compared to patients admitted during the previous waves, and similar to the first wave. This suggest that COVID-19, even though it is less severe, continues to be a challenging problem both for patients and clinicians, requiring attention both for monitoring and care, especially in fragile patients with multiple comorbidities, being CKD a possible strong marker of this fragility. Taking account of the pivotal role of CKD that emerged from our analysis, more studies are needed for a better management of COVID-19 in patients with CKD. Conclusively, it is essential to proceed to collect more epidemiological and clinical data on COVID-19, in order to comprehend its evolution and update the clinical guidelines to respond to this evolving disease based on the emerging priorities.

## Data availability statement

The original contributions presented in the study are included in the article/supplementary material, further inquiries can be directed to the corresponding author.

## Ethics statement

The studies involving humans were approved by ethical committee of the Calabria Region (protocol reference: FESR/FSE 2014–2020 DDRC n. 4585, Action 10.5.12, noCOVID19@UMG). The studies were conducted in accordance with the local legislation and institutional requirements. Written informed consent for participation was not required from the participants or the participants’ legal guardians/next of kin in accordance with the national legislation and institutional requirements.

## Author contributions

ET: Conceptualization, Data curation, Formal analysis, Supervision, Validation, Writing – original draft, Writing – review & editing. VO: Conceptualization, Data curation, Supervision, Validation, Writing – original draft, Writing – review & editing. CD: Conceptualization, Data curation, Supervision, Validation, Writing – original draft, Writing – review & editing. SR: Supervision, Writing – review & editing. FS: Data curation, Writing – original draft. RL: Data curation, Writing – original draft. BT: Data curation, Writing – original draft. VG: Data curation, Writing – original draft. PF: Data curation, Writing – original draft. AR: Supervision, Validation, Writing – review & editing. MB: Data curation, Formal analysis, Supervision, Validation, Writing – review & editing. CT: Conceptualization, Data curation, Formal analysis, Supervision, Validation, Writing – original draft, Writing – review & editing.

## IDTM UMG COVID-19 group

In addition to the authors listed above, IDTM UMG COVID-19 Group included: Chiara Costa, Helen Morrone, Maria Teresa Tassone, Lavinia Berardelli, Riccardo Serraino, Gabriele Bruno, Sara Palma Gullì, Francesco Romeo, Simona Mongiardi, Rita Pallone, Rita Laudari, Alessandro Sanfilippo, Francesco Saverio Costanzo, Daniela Patrizia Foti, Giovanni Matera, Federico Longhini, Andrea Bruni, Eugenio Garofalo, Eugenio Biamonte, Domenico Laganà, Maria Petullà, Bernardo Bertucci, Angela Quirino, Giorgio Settimo Barreca, Aida Giancotti, Luigia Gallo, Angelo Lamberti, Nadia Marascio, Adele Emanuela De Francesco.

## References

[1] Available at: https://www.epicentro.iss.it/coronavirus/sars-cov-2-dashboard.

[2] Sacerdote C Milani L Castiglione A Pagano E Migliore E Albera C. Risk of intensive care unit admission or mortality in patients hospitalised for COVID-19 during the first two waves: an Italian cohort study. J Infect. (2022) 85:436–80. doi: 10.1016/j.jinf.2022.06.023, PMID: 35781015 PMC9245397

[3] Pisaturo M Russo A Pattapola V Astorri R Maggi P Numis FG. Clinical characterization of the three waves of COVID-19 occurring in southern Italy: results of a multicenter cohort study. Int J Environ Res Public Health. (2022) 19:16003. doi: 10.3390/ijerph192316003, PMID: 36498078 PMC9738780

[4] Giacomelli A Ridolfo AL Pezzati L Oreni L Carrozzo G Beltrami M. Mortality rates among COVID-19 patients hospitalised during the first three waves of the epidemic in Milan, Italy: a prospective observational study. PLoS One. (2022) 17:e0263548. doi: 10.1371/journal.pone.0263548, PMID: 35404963 PMC9000097

[5] Buscemi S Davoli C Trecarichi EM Morrone HL Tassone B Buscemi C. The three facets of the SARS-CoV-2 pandemic during the first two waves in the northern, central, and southern Italy. J Infect Public Health. (2023) 16:520–5. doi: 10.1016/j.jiph.2023.02.002, PMID: 36801631 PMC9902343

[6] Zuccaro V Colaneri M Asperges E Valsecchi P Sambo M Maiocchi L. Mortality due to COVID-19 during the pandemic: a comparison of first, secondo and third SMAtteo COvid19 REgistry (SMACORE). Heliyon. (2022) 8:e08895. doi: 10.1016/j.heliyon.2022.e08895, PMID: 35132388 PMC8810275

[7] Trecarichi EM Mazzitelli M Serapide F Pelle MC Tassone B Arrighi E. Clinical characteristics and predictors of mortality associated with COVID-19 in elderly patients from a long-term care facility. Sci Rep. (2020) 10:20834. doi: 10.1038/s41598-020-77641-7, PMID: 33257703 PMC7705720

[8] Ticinesi A Parise A Nouvenne A Cerundolo N Prati B Guerra A. Insights from comparison of the clinical presentation and outcomes of patients hospitalized with COVID-19 in an Italian internal medicine ward during first and third wave. Front Med (Lausanne). (2023) 10:1112728. doi: 10.3389/fmed.2023.1112728, PMID: 36817786 PMC9928966

[9] Mesle MM Brown J Mook P Hagan J Pastore R Bundle N. Estimated number of deaths directly averted in people 60 years and older as a result of COVID-19 vaccination in the WHO European region, December 2020 to November 2021. Euro Surveill. (2021) 26. doi: 10.2807/1560-7917.ES.2021.26.47.2101021 PMC8619871 34823641

[10] Leidi F Boari GEM Scarano O Mangili B Gorla G Corbani A. Comparison of the characteristics, morbidity and mortality of COVID-19 between first and second/third wave in a hospital setting in Lombardy: a retrospective cohort study. Intern Emerg Med. (2022) 17:1941–9. doi: 10.1007/s11739-022-03034-5, PMID: 35809152 PMC9521559

[11] Aznar-Gimeno R Pano-Pardo JR Esteban LM Labata-Lezaun G Esquillor-Rodrigo MJ Lanas A. Changes in severity, mortality, and virus genome among a Spanish cohort of patients hospitalized with SARS-CoV-2. Sci Rep. (2021) 11:18844. doi: 10.1038/s41598-021-98308-x, PMID: 34552127 PMC8458298

[12] Buttenschon HN Lynggaard V Sandbol SG Glassou EN Haagerup A. Comparison of the clinical presentation across two waves of COVID-19: a retrospective cohort study. BMC Infect Dis. (2022) 22:423. doi: 10.1186/s12879-022-07413-3, PMID: 35505306 PMC9063242

[13] Lampl BMJ Edenharter B Leitzmann MF Salzberger B. COVID-19-related deaths: a 2-year inter-wave comparison of mortality data from Germany. Infection. (2023) 51:1147–52. doi: 10.1007/s15010-023-01982-4, PMID: 36690889 PMC9870770

[14] Fois SS Zinellu E Zinellu A Merella M Pau MC Carru C. Comparison of clinical features, complete blood count parameters, and outcomes between two distinct waves of COVID-19: a monocentric report from Italy. Healthcare (Basel). (2022) 10:2427. doi: 10.3390/healthcare10122427 36553950 PMC9778399

[15] Westreich D Greenland S. The table 2 fallacy: presenting and interpreting confounder and modifier coefficients. Am J Epidemiol. (2013) 177:292–8. doi: 10.1093/aje/kws412, PMID: 23371353 PMC3626058

[16] Lapointe-Shaw L Bouck Z Howell NA Lange T Orchanian-Cheff A Austin PC. Mediation analysis with a time-to-event outcome: a review of use and reporting in healthcare research. BMC Med Res Methodol. (2018) 18:118. doi: 10.1186/s12874-018-0578-7, PMID: 30373524 PMC6206666

[17] Hothorn T Hornik K Zeileis A. Unbiased recursive partitioning: a conditional inference framework. J Comput Graph Stat. (2006) 15:651–74. doi: 10.1198/106186006X133933

[18] Zhang SX Arroyo Marioli F Gao R Wang S. A second wave? What do people mean by COVID waves? A working definition of epidemic waves. Risk Manag Healthc Policy. (2021) 14:3775–82. doi: 10.2147/RMHP.S326051, PMID: 34548826 PMC8448159

[19] Characterisation WHOWGotC, Management of C-i. A minimal common outcome measure set for COVID-19 clinical research. Lancet Infect Dis. (2020) 20:e192–7. doi: 10.1016/S1473-3099(20)30483-7 32539990 PMC7292605

[20] World Medical A. World medical association declaration of Helsinki: ethical principles for medical research involving human subjects. JAMA. (2013) 310:2191–4. doi: 10.1001/jama.2013.281053 24141714

[21] Therneau T. A package for survival analysis in R. R package version 3.3 (2022).

[22] R RCT. A language and environment for statistical computing. Vienna, Austria: R Foundation for Statistical Computing (2022).

[23] Provenzano M Rotundo S Chiodini P Gagliardi I Michael A Angotti E. Contribution of predictive and prognostic biomarkers to clinical research on chronic kidney disease. Int J Mol Sci. (2020) 21:5846. doi: 10.3390/ijms21165846, PMID: 32823966 PMC7461617

[24] Hall VG Solera JT Al-Alahmadi G Marinelli T Cardinal H Poirier C. Severity of COVID-19 among solid organ transplant recipients in Canada, 2020-2021: a prospective, multicentre cohort study. CMAJ. (2022) 194:E1155–63. doi: 10.1503/cmaj.220620, PMID: 36302101 PMC9435532

[25] Bhaskaran K Bacon S Evans SJ Bates CJ Rentsch CT MacKenna B. Factors associated with deaths due to COVID-19 versus other causes: population-based cohort analysis of UK primary care data and linked national death registrations within the OpenSAFELY platform. Lancet Reg Health Eur. (2021) 6:100109. doi: 10.1016/j.lanepe.2021.100109, PMID: 33997835 PMC8106239

[26] Henrard S Speybroeck N Hermans C. Classification and regression tree analysis vs. multivariable linear and logistic regression methods as statistical tools for studying haemophilia. Haemophilia. (2015) 21:715–22. doi: 10.1111/hae.12778, PMID: 26248714

[27] D'Arminio Monforte A Tavelli A Bai F Tomasoni D Falcinella C Castoldi R. Declining mortality rate of hospitalised patients in the second wave of the COVID-19 epidemics in Italy: risk factors and the age-specific patterns. Life (Basel). (2021) 11:979. doi: 10.3390/life11090979 34575128 PMC8464683

[28] Polverino F Stern DA Ruocco G Balestro E Bassetti M Candelli M. Comorbidities, cardiovascular therapies, and COVID-19 mortality: a Nationwide, Italian observational study (ItaliCO). Front Cardiovasc Med. (2020) 7:585866. doi: 10.3389/fcvm.2020.585866, PMID: 33195473 PMC7583635

[29] Di Castelnuovo A Bonaccio M Costanzo S Gialluisi A Antinori A Berselli N. Common cardiovascular risk factors and in-hospital mortality in 3,894 patients with COVID-19: survival analysis and machine learning-based findings from the multicentre Italian CORIST study. Nutr Metab Cardiovasc Dis. (2020) 30:1899–913. doi: 10.1016/j.numecd.2020.07.031, PMID: 32912793 PMC7833278

[30] Chenchula S Karunakaran P Sharma S Chavan M. Current evidence on efficacy of COVID-19 booster dose vaccination against the omicron variant: a systematic review. J Med Virol. (2022) 94:2969–76. doi: 10.1002/jmv.27697, PMID: 35246846 PMC9088621

[31] Najjar-Debbiny R Gronich N Weber G Khoury J Amar M Stein N. Effectiveness of Paxlovid in reducing severe coronavirus disease 2019 and mortality in high-risk patients. Clin Infect Dis. (2023) 76:e342–9. doi: 10.1093/cid/ciac443, PMID: 35653428 PMC9214014

[32] Mazzitelli M Trunfio M Sasset L Scaglione V Ferrari A Mengato D. Risk of hospitalization and sequelae in patients with COVID-19 treated with 3-day early remdesivir vs. controls in the vaccine and omicron era: a real-life cohort study. J Med Virol. (2023) 95:e28660. doi: 10.1002/jmv.28660, PMID: 36905216

[33] Menni C Valdes AM Polidori L Antonelli M Penamakuri S Nogal A. Symptom prevalence, duration, and risk of hospital admission in individuals infected with SARS-CoV-2 during periods of omicron and delta variant dominance: a prospective observational study from the ZOE COVID study. Lancet. (2022) 399:1618–24. doi: 10.1016/S0140-6736(22)00327-0, PMID: 35397851 PMC8989396

[34] Petrone D Mateo-Urdiales A Sacco C Riccardo F Bella A Ambrosio L. Reduction of the risk of severe COVID-19 due to omicron compared to Delta variant in Italy (November 2021—February 2022). Int J Infect Dis. (2023) 129:135–41. doi: 10.1016/j.ijid.2023.01.027, PMID: 36708869 PMC9877142

[35] Rotundo S Borelli M Scaglione V Lionello R Biamonte F Olivadese V. Interleukin-6(2)/lymphocyte as a proposed predictive index for COVID-19 patients treated with monoclonal antibodies. Clin Exp Med. (2023) 23:3681–7. doi: 10.1007/s10238-023-01081-6 37097384 PMC10127195

[36] Biamonte F Botta C Mazzitelli M Rotundo S Trecarichi EM Foti D. Combined lymphocyte/monocyte count, D-dimer and iron status predict COVID-19 course and outcome in a long-term care facility. J Transl Med. (2021) 19:79. doi: 10.1186/s12967-021-02744-2, PMID: 33596963 PMC7887565

[37] La Torre G Marte M Massetti AP Carli SM Romano F Mastroianni CM. The neutrophil/lymphocyte ratio as a prognostic factor in COVID-19 patients: a case-control study. Eur Rev Med Pharmacol Sci. (2022) 26:1056–64. doi: 10.26355/eurrev_202202_28017, PMID: 35179773

[38] Mimmi S Zimbo AM Rotundo S Cione E Nistico N Aloisio A. SARS CoV-2 spike protein-guided exosome isolation facilitates detection of potential miRNA biomarkers in COVID-19 infections. Clin Chem Lab Med. (2023) 61:1518–24. doi: 10.1515/cclm-2022-1286, PMID: 36972680

[39] Kimura Y Cristancho-Rojas CN Kimura-Sandoval Y Tapia-Sosa R Guerrero-Torres L Licano-Zubiate M. Prognostic utility of the chest computed tomography severity score for the requirement of mechanical ventilation and mortality in hospitalized patients with COVID-19. Heliyon. (2023) 9:e16020. doi: 10.1016/j.heliyon.2023.e16020, PMID: 37153411 PMC10151249

[40] Demelo-Rodriguez P Galeano-Valle F Ordieres-Ortega L Siniscalchi C Martin Del Pozo M Fidalgo A. Validation of a prognostic score to identify hospitalized patients with COVID-19 at increased risk for bleeding. Viruses. (2021) 13:2278. doi: 10.3390/v13112278, PMID: 34835085 PMC8621368

[41] Tan BWL Tan BWQ Tan ALM Schriver ER Gutierrez-Sacristan A Das P. Long-term kidney function recovery and mortality after COVID-19-associated acute kidney injury: an international multi-Centre observational cohort study. EClinicalMedicine. (2023) 55:101724. doi: 10.1016/j.eclinm.2022.101724, PMID: 36381999 PMC9640184

[42] Almeida NBF Filgueiras PS Lourenco AJ Bicalho CMF Corsini CA de Miranda DAP. COVID-19 in patients with chronic kidney disease: a 2-year study of incidence and mortality in relation to the variants of concern waves in Brazil. Trans R Soc Trop Med Hyg. (2023) 117:606–8. doi: 10.1093/trstmh/trad019, PMID: 37042271

[43] Torti C Olimpieri PP Bonfanti P Tascini C Celant S Tacconi D. Real-life comparison of mortality in patients with SARS-CoV-2 infection at risk for clinical progression treated with molnupiravir or nirmatrelvir plus ritonavir during the omicron era in Italy: a nationwide, cohort study. Lancet Reg Health Eur. (2023) 31:100684. doi: 10.1016/j.lanepe.2023.100684, PMID: 37547273 PMC10398591

[44] Regnery R Tappero J. Unraveling mysteries associated with cat-scratch disease, bacillary angiomatosis, and related syndromes. Emerg Infect Dis. (1995) 1:16–21. doi: 10.3201/eid0101.950103 8903149 PMC2626823

[45] De Marco C Veneziano C Massacci A Pallocca M Marascio N Quirino A. Dynamics of viral infection and evolution of SARS-CoV-2 variants in the Calabria area of southern Italy. Front Microbiol. (2022) 13:934993. doi: 10.3389/fmicb.2022.934993, PMID: 35966675 PMC9366435

[46] Veneziano C Marascio N De Marco C Quaresima B Biamonte F Trecarichi EM. The spread of SARS-CoV-2 omicron variant in CALABRIA: a Spatio-temporal report of viral genome evolution. Viruses. (2023) 15:408. doi: 10.3390/v15020408, PMID: 36851622 PMC9963258

[47] Kundi M. Vaccine effectiveness against delta and omicron variants of SARS-CoV-2. BMJ. (2023) 381:1111. doi: 10.1136/bmj.p1111 37220942

[48] Wang Y Teng F Wang M Li S Lin Y Cai H. Monitoring spatiotemporal distribution of the GDP of major cities in China during the COVID-19 pandemic. Int J Environ Res Public Health. (2022) 19:8048. doi: 10.3390/ijerph19138048 35805721 PMC9265774

